# A case report of pathological complete response in an elderly patient with advanced urothelial carcinoma treated with disitamab vedotin plus toripalimab

**DOI:** 10.1186/s12894-026-02194-2

**Published:** 2026-06-17

**Authors:** Liang Songlin, Liu Yunxun, He Peiyu, Hu Wen, Li Mingyong

**Affiliations:** Urology department, The First Affiliated Hospital, Hengyang Medical School, University of South China, Hengyang, China

**Keywords:** Urothelial carcinoma, Antibody–drug conjugate, Immunotherapy, Case report, Complete response

## Abstract

Urothelial carcinoma is a common malignancy of the urinary tract and occurs predominantly in elderly men. Although surgery, chemotherapy, and radiotherapy remain the main treatment options, their efficacy in advanced disease is often limited, particularly in elderly patients with comorbidities or poor surgical tolerance. Antibody-drug conjugates (ADCs) combined with immune checkpoint inhibitors have recently emerged as a promising therapeutic strategy. We report the case of a 79-year-old man with advanced urothelial carcinoma who declined radical cystectomy and received disitamab vedotin plus toripalimab. Histopathological examination confirmed high-grade muscle-invasive urothelial carcinoma, and supplementary immunohistochemistry showed HER2 expression of 1+. FISH/ISH and PD-L1 testing were not performed. The patient received treatment every 3 weeks over 2 consecutive days, with disitamab vedotin on day 1 and toripalimab on day 2. After 6 cycles, imaging showed marked tumor regression, and repeat transurethral resection of the tumor bed revealed no residual tumor cells, consistent with pathological complete response. The treatment was generally well tolerated, with only mild fatigue and nausea. This case suggests that disitamab vedotin combined with toripalimab may have activity in selected patients with advanced urothelial carcinoma, including those with low HER2 expression. However, the findings should be interpreted cautiously, and further studies are needed to clarify the role of this regimen and the value of predictive biomarkers.

**Supplementary Information:**

The online version contains supplementary material available at 10.1186/s12894-026-02194-2.

Bladder cancer is one of the most common malignancies of the urinary system, and more than 90% of cases are urothelial carcinoma. It occurs predominantly in elderly individuals, especially men. For patients with muscle-invasive or locally advanced disease, radical surgery and platinum-based chemotherapy remain the main components of treatment. However, in real-world practice, a proportion of elderly patients are not ideal candidates for radical cystectomy or intensive systemic therapy because of frailty, comorbidities, organ dysfunction, or personal refusal of surgery. Antibody-drug conjugates have introduced a new therapeutic option for urothelial carcinoma by combining antigen-directed targeting with delivery of potent cytotoxic payloads. At the same time, immune checkpoint inhibitors have shown activity in advanced urothelial carcinoma. The combination of these two approaches has attracted increasing attention, particularly in patients who may not tolerate conventional treatment well. Here, we report an elderly patient with advanced urothelial carcinoma who declined radical cystectomy and achieved pathological complete response after treatment with disitamab vedotin plus toripalimab. The case is of interest because the tumor showed HER2 immunohistochemistry of 1+, and the patient achieved a deep response without surgical removal of the bladder.

## Clinical data

**Fig. 1:**
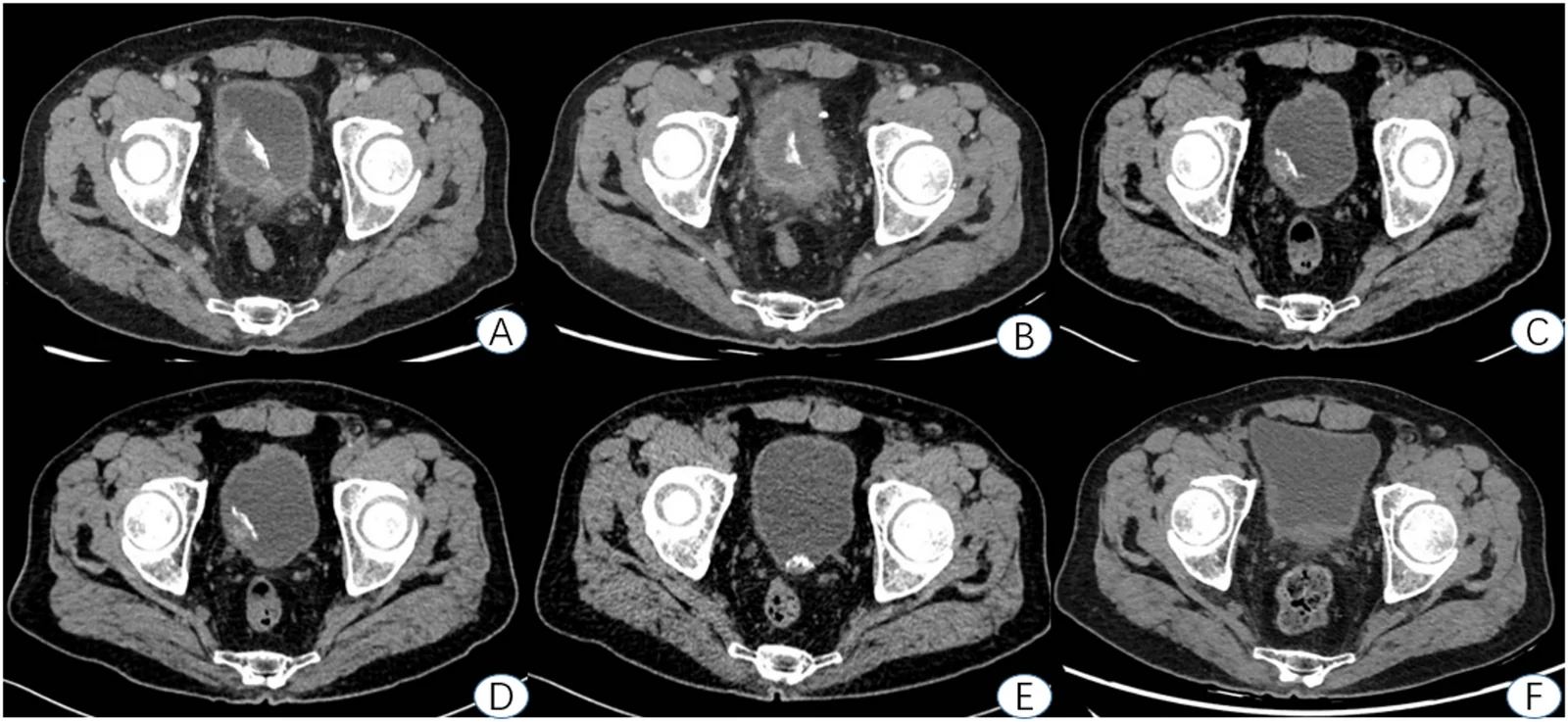
Serial CTU findings during treatment. **A**: Baseline CTU showing a bladder mass measuring approximately 67 mm at maximum diameter.** B–C**: Tumor shrinkage after 3 cycles of treatment. **D–E**: Further reduction of the lesion to approximately 2 cm after 6 cycles.** F**: No obvious recurrence on CTU one month after completion of treatment

**Fig. 2:**
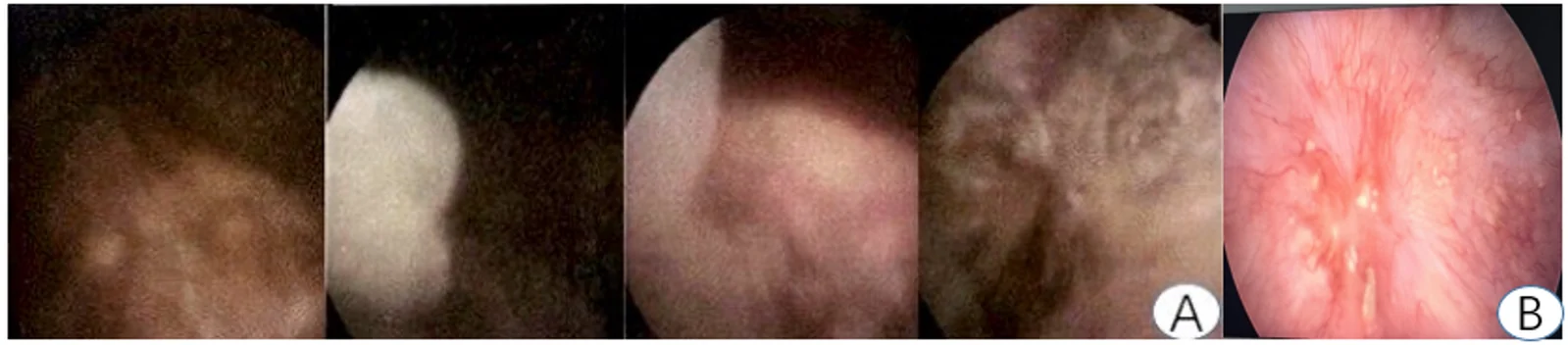
Cystoscopic findings before and after treatment. **A**: Initial cystoscopy showing a broad-based cauliflower-like tumor on the right lateral bladder wall with calcification at the base.** B**: Cystoscopic re-evaluation after 6 cycles showing a scar-like lesion with slight calcification

**Fig. 3:**
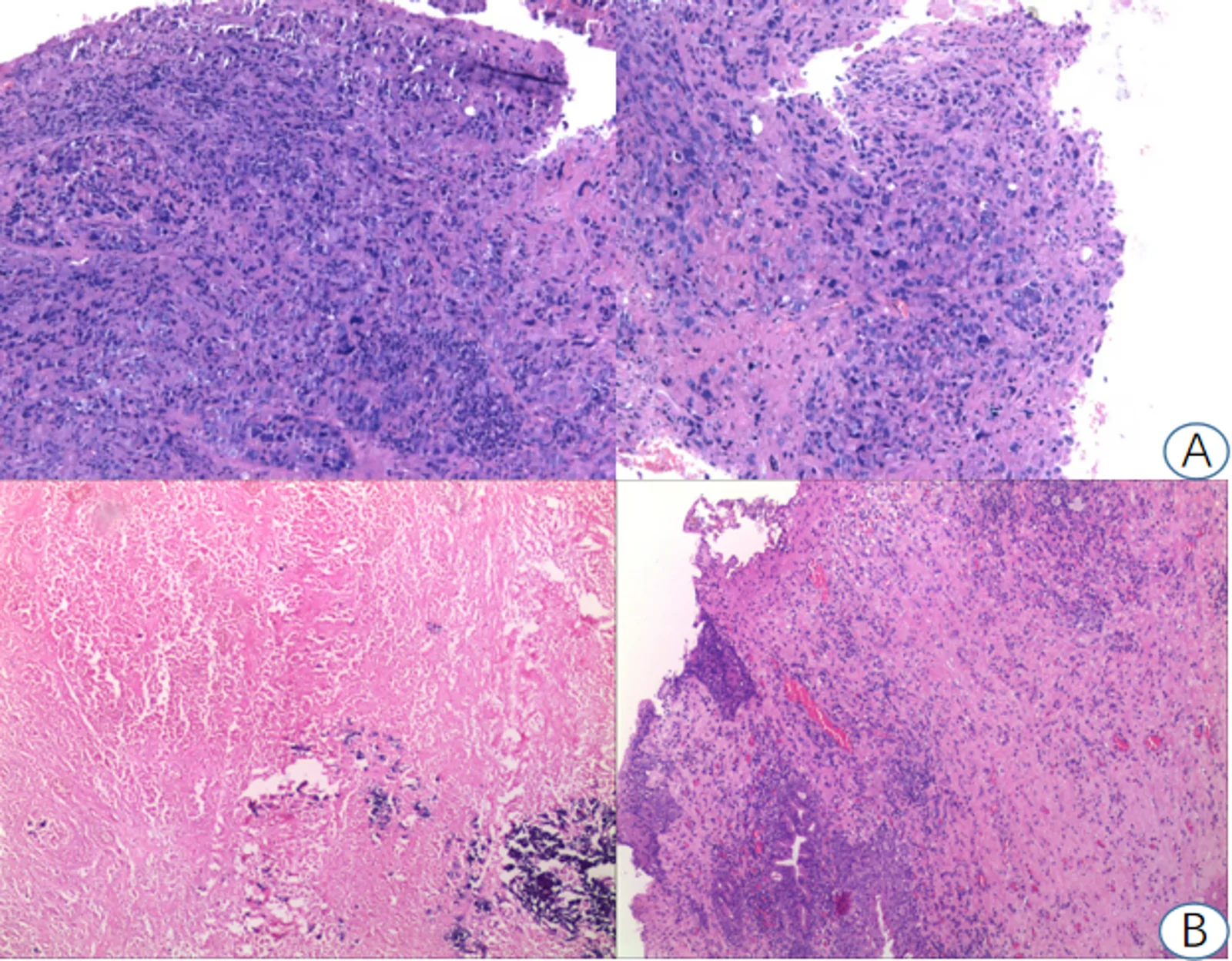
Pathological findings before and after treatment. **A**: Initial pathological specimen showing high-grade invasive urothelial carcinoma with HER2 immunohistochemistry scored as 1+. **B**: Repeat pathological examination after 6 cycles showing no residual viable tumor cells, consistent with pathological complete response

A 79-year-old man was admitted in January 2024 with a 2-day history of painless gross hematuria. Urinalysis showed gross hematuria with proteinuria (2 g/L). Computed tomography urography (CTU) revealed an irregular soft-tissue mass on the right posterior wall of the bladder, measuring approximately 67 × 39 × 56 mm, with right hydronephrosis and multiple enlarged pelvic lymph nodes suggestive of nodal metastases (Fig. 1A). The lesion showed an indistinct boundary with the right seminal vesicle and prostate, raising suspicion of adjacent invasion. Cystoscopy demonstrated a broad-based cauliflower-like tumor on the right lateral bladder wall with calcification at the base (Fig. 2A). Histopathological examination confirmed high-grade muscle-invasive urothelial carcinoma with necrosis. A supplementary pathology report dated February 3, 2024 showed HER2 immunohistochemistry scored as 1+ (Fig. 3A). FISH/ISH testing was not performed, and PD-L1 expression was not assessed. The patient also had several comorbidities, including bilateral lower-extremity venous thrombosis, benign pulmonary nodules, old rib fractures, aortic calcification, and hypertension. The patient had a history of bilateral lower-extremity venous thrombosis. Anticoagulation with nadroparin was documented during an earlier hospitalization, whereas no anticoagulation therapy was documented in the later available records. No formal DVT risk stratification or ECOG performance status was documented in the available medical records. Baseline laboratory evaluation showed mildly impaired renal function, with serum creatinine remaining near the upper limit of normal and an estimated glomerular filtration rate (eGFR) of approximately 66 mL/min. Liver function parameters were within normal limits, and coagulation tests were largely unremarkable. Based on the imaging findings, the disease was clinically staged as cT3N2M0. However, because the lesion was reported to have an indistinct boundary with the right seminal vesicle, local staging should be interpreted with caution, as it was based on imaging rather than surgical pathology. The patient declined radical cystectomy. Considering the patient’s age, comorbidities, treatment tolerance, and financial situation, systemic therapy with disitamab vedotin plus toripalimab was initiated. One treatment cycle was administered every 3 weeks over 2 consecutive days. On day 1, disitamab vedotin 120 mg was intravenously infused in 250 mL normal saline over at least 60 min, with dexamethasone given prior to infusion. On day 2, toripalimab 240 mg was intravenously administered in 100 mL normal saline using a dedicated infusion set over at least 60 min. The patient’s body weight was recorded as 66 kg in the available medical records. Based on this, the administered fixed dose of 120 mg disitamab vedotin corresponded to approximately 1.8 mg/kg, which is generally consistent with commonly used dosing in clinical studies. This dosing was consistent with commonly reported regimens in clinical studies. However, a fixed dose of 120 mg disitamab vedotin would approximately correspond to 2.0 mg/kg in a patient weighing around 60 kg, suggesting that the administered dose was broadly in line with commonly used study dosing. After 3 cycles, gross hematuria resolved and the tumor had decreased in size to approximately 4 cm (Fig. 1B, C). After 6 cycles, the patient experienced mild fatigue and nausea during treatment. CT reexamination showed that the mass had shrunk to approximately 2 cm (Fig. 1D, E), and cystoscopy revealed a scar-like appearance with slight calcification (Fig. 2B). Extensive electroresection of the original tumor bed area down to the muscular layer was then performed for treatment response assessment. Pathological examination showed no residual viable tumor cells, consistent with pathological complete response (Fig. 3B). One month later, repeat CTU showed no evidence of obvious recurrence (Fig. 1F). Serial laboratory assessments during treatment and follow-up did not reveal clinically significant hepatotoxicity, and renal function remained relatively stable, with eGFR declining slightly from approximately 66 mL/min to 59 mL/min without obvious clinical deterioration. At the latest follow-up in June 2025, no radiological progression or clinical recurrence was observed, and the patient remained under regular surveillance.

## Discussion

This case describes an elderly patient with advanced urothelial carcinoma who declined radical cystectomy and achieved a deep response after treatment with disitamab vedotin plus toripalimab. In elderly patients with advanced bladder cancer, treatment decisions are often influenced by comorbidities and limited tolerance to standard therapies [1, 2]. In this context, achieving a deep response with a non-surgical approach is clinically meaningful.

At the same time, the present case should be interpreted within the context of currently available evidence rather than as proof of efficacy on its own. Early studies have shown antitumor activity of disitamab vedotin in previously treated HER2-expressing urothelial carcinoma [3, 4], Subsequent clinical studies have further confirmed its efficacy and safety profile [5]. More recently, combination therapy with disitamab vedotin and toripalimab has shown encouraging response rates, including complete responses in a subset of patients [6]. The outcome in our patient is broadly consistent with those observations, but this report mainly provides a real-world clinical example rather than definitive new evidence.The main point of interest in this case is that the patient had low HER2 expression, with supplementary immunohistochemistry showing HER2 1+, and still achieved pathological complete response after combination therapy. This HER2 finding is important, but it should be interpreted cautiously. In the earlier version of the manuscript, “HER2 positive” or “HER2 low” was described too loosely. Based on the supplementary pathology report, the more accurate wording in this case is HER2 immunohistochemistry 1+. However, FISH/ISH was not performed, so the HER2 status was not further characterized at the gene amplification level. In addition, HER2 assessment in urothelial carcinoma is not yet as standardized as it is in breast or gastric cancer, and its predictive value remains uncertain [4, 7]. Therefore, it would be inappropriate to make a strong claim that this case confirms efficacy in a biologically defined HER2-low subgroup. A more balanced interpretation is that this case suggests possible activity of the regimen in a patient whose tumor showed HER2 1 + staining.Another point that requires clarification is how treatment response was assessed. This observation is noteworthy in the context of limited evidence regarding treatment efficacy in tumors with low HER2 expression. In this patient, complete remission was not defined by cystectomy pathology, but by serial imaging, cystoscopic findings, and repeat resection of the prior tumor bed. Therefore, the most accurate description is marked radiological regression followed by pathological complete response on a repeat TURBT specimen. This is an encouraging result, but it is not entirely equivalent to pT0 status on radical cystectomy. The possibility of sampling limitation should therefore be acknowledged [2, 9]. Even so, the absence of viable tumor cells in the resected tumor bed remains an important finding and should be emphasized rather than described simply as “tumor necrosis.” The treatment schedule was administered over 2 consecutive days every 3 weeks. The dosing strategy also warrants clarification. According to the medical records, one cycle was administered every 3 weeks over 2 consecutive days, with disitamab vedotin given on day 1 and toripalimab on day 2. This clarifies that the two agents were used sequentially within each cycle rather than as an indistinct combined infusion schedule. The dosing strategy also deserves explanation. Disitamab vedotin was administered at a fixed dose of 120 mg per cycle. Although the patient’s exact body weight was not retrievable, this dose corresponded to approximately 1.8 mg/kg based on the patient’s recorded body weight [4, 6]. Clinically, the regimen was generally well tolerated, with no treatment discontinuation due to adverse events. Serial laboratory assessments demonstrated stable organ function, with no clinically relevant hepatotoxicity and no significant deterioration in renal function during treatment and follow-up.This safety profile appears broadly consistent with previous reports of ADC-based treatment in urothelial carcinoma [4, 6]. From a mechanistic perspective, the activity of this combination is biologically plausible. Disitamab vedotin not only delivers a cytotoxic payload to HER2-expressing tumor cells, but may also promote immunogenic cell death and alter the tumor microenvironment in ways that enhance antitumor immunity. Immune checkpoint inhibition may further augment this effect by restoring T-cell activity. Although these mechanisms were not directly examined in the present case, they are supported by the current understanding of ADC biology and combination strategies involving ADCs and immunotherapy [7, 8, 10]. Accordingly, the discussion should remain restrained. Rather than claiming mechanistic confirmation, it is more appropriate to state that the observed response is compatible with a possible synergistic effect between ADC therapy and immune checkpoint blockade. This report also has several limitations. First, it is a single case with limited follow-up, and durable bladder preservation cannot yet be assumed [9]. Second, clinical staging was based on imaging; although the patient was classified as cT3N2M0, the reported proximity to or possible invasion of the seminal vesicle means that local staging should be interpreted carefully [2]. Third, key biomarkers were incomplete: HER2 was reported only as IHC 1+, while FISH/ISH and PD-L1 testing were not performed. Finally, because this was not a surgical series, the findings cannot be used to support broad conclusions about replacing current standard therapy. In addition, the patient had a history of bilateral lower-extremity venous thrombosis, but detailed anticoagulation management and its potential impact on treatment decisions could not be fully reconstructed from the available records.

Overall, this case suggests that disitamab vedotin plus toripalimab may induce a deep response in selected patients with advanced urothelial carcinoma who are not ideal candidates for radical surgery or conventional chemotherapy [6]. The observation is particularly interesting because the tumor showed HER2 1 + staining. Nevertheless, the result should be regarded as hypothesis-generating rather than practice-changing. Further prospective studies with standardized biomarker assessment, clearer response criteria, and longer follow-up are needed before broader conclusions can be drawn [10].

## Conclusion

This case suggests that disitamab vedotin plus toripalimab may be a potentially active treatment option for selected elderly patients with advanced urothelial carcinoma who are not suitable for radical cystectomy. In this patient, the regimen was associated with marked radiological regression and pathological complete response on repeat transurethral resection, despite low HER2 expression by immunohistochemistry. However, because this is a single case with incomplete biomarker assessment and limited follow-up, the findings should be interpreted cautiously. Further studies are needed to define the efficacy of this combination and to clarify the role of predictive biomarkers in patient selection.

## Supplementary Information

Supplementary Material 1.

## Data Availability

All data analyzed and presented in this case report are contained within the main text, figures, and supplementary materials of this article. Raw clinical data supporting the conclusions of this study (such as anonymized copies of the patient’s imaging studies) may be made available upon reasonable request to the corresponding author, subject to appropriate ethical approval. The complete raw data from the imaging and pathological examinations cannot be publicly disclosed due to concerns regarding patient privacy.
