# Supplementary material for: A case report of pathological complete response in an elderly patient with advanced urothelial carcinoma treated with disitamab vedotin plus toripalimab
Source: BMC Urol. 2026 Jun 17;26:210. doi: 10.1186/s12894-026-02194-2 (PMC13508225; doi:10.1186/s12894-026-02194-2)
Supplement: Supplementary file 1 [file 12894_2026_2194_MOESM1_ESM.pdf]

CARE case report guidelines

CARE Checklist of information to include when writing a case re[port]

(cc) BY-NC-ND

Topic	Item	Checklist item description	Reported on Line
Title	1	The diagnosis or intervention of primary focus followed by the words "case report"	1
Key Words	2	2 to 5 key words that identify diagnoses or interventions in this case report, including "case report"	20-21
Abstract (no references)	3a	Introduction: What is unique about this case and what does it add to the scientific literature?	24-39
	3b	Main symptoms and/or important clinical findings	41-42
	3c	The main diagnoses, therapeutic interventions, and outcomes	53-65
	3d	Conclusion—What is the main "take-away" lesson(s) from this case?	136-151
Introduction	4	One or two paragraphs summarizing why this case is unique (**may include references**)	7-20
Patient Information	5a	De-identified patient specific information.	40-41
	5b	Primary concerns and symptoms of the patient.	41-42
	5c	Medical, family, and psycho-social history including relevant genetic information	50-52
	5d	Relevant past interventions with outcomes	52-53
Clinical Findings	6	Describe significant physical examination (PE) and important clinical findings.	42-50
Timeline	7	Historical and current information from this episode of care organized as a timeline	56-65
Diagnostic Assessment	8a	Diagnostic testing (such as PE, laboratory testing, imaging, surveys).	42-50
	8b	Diagnostic challenges (such as access to testing, financial, or cultural)	54-55
	8c	Diagnosis (including other diagnoses considered)	52-53
	8d	Prognosis (such as staging in oncology) where applicable	53-54
Therapeutic Intervention	9a	Types of therapeutic intervention (such as pharmacologic, surgical, preventive, self-care)	55-56
	9b	Administration of therapeutic intervention (such as dosage, strength, duration)	56
	9c	Changes in therapeutic intervention (with rationale)	not mentioned
Follow-up and Outcomes	10a	Clinician and patient-assessed outcomes (if available)	58-65
	10b	Important follow-up diagnostic and other test results	60-6 5
	10c	Intervention adherence and tolerability (How was this assessed?)	59-60
	10d	Adverse and unanticipated events	not mentioned
Discussion	11a	A scientific discussion of the strengths AND limitations associated with this case report	140-146
	11b	Discussion of the relevant medical literature **with references**.	192-253
	11c	The scientific rationale for any conclusions (including assessment of possible causes)	146-151
	11d	The primary "take-away" lessons of this case report (without references) in a one paragraph conclusion	152-161
Patient Perspective	12	The patient should share their perspective in one to two paragraphs on the treatment(s) they received	do not contian
Informed Consent	13	Did the patient give informed consent? Please provide if requested	Yes ☑ No ☐
